# Sea Medick Volatile Oil: Antioxidant, Insecticidal, In Vitro Cytotoxic Activity on SAF‐1 Cells and Anti‐Lipoxygenase Assessment via In Vitro and In Silico Methods

**DOI:** 10.1002/fsn3.72373

**Published:** 2026-09-24

**Authors:** Marwa Melliti, Marcello Iriti, Mabrouk Horchani, Ikbel Chaieb, Cristóbal Espinosa‐Ruiz, Maria Angeles Esteban, Hayet Edziri

**Affiliations:** ^1^ Laboratory of Transmissible Diseases and Biologically Active Substances (LR99 ES27), Faculty of Pharmacy University of Monastir Monastir Tunisia; ^2^ Department of Biomedical, Surgical and Dental Sciences University of Milan Milan Italy; ^3^ Laboratory of Heterocyclic Chemistry, Natural Products and Reactivity (LR11Es39), Medicinal Chemistry and Natural Products, Faculty of Sciences of Monastir University of Monastir Monastir Tunisia; ^4^ Laboratory of Production and Protection for a Sustainable Horticulture (LR21AGR03), Regional Centre of Research on Horticulture and Organic Agriculture University of Sousse Sousse Tunisia; ^5^ Biotechnology and Aquaculture Laboratory Instituto Murciano de Investigación y Desarrollo Agrarioy Medioambiental Murcia Spain; ^6^ Immunobiology for Aquaculture Group, Department of Cell Biology and Histology, Faculty of Biology University of Murcia Murcia Spain

**Keywords:** anti‐lipoxygenase, antioxidants, cytotoxicity, essential oil, insecticide, *Medicago marina*

## Abstract

The Mediterranean plant *Medicago marina* L., native to North Africa, Spain, and Central Asia, is instrumental in the stabilization of coastal dunes and holds considerable economic significance. In addition to the plant's insecticidal potential against *Tribolium castaneum* , anti‐lipoxygenase activity, and cytotoxic activity against SAF‐1 cell lines, this study evaluated the antioxidant capacity of the essential oil, whose chemical composition was previously published in our paper. The observed antioxidant effect may be related to the reducing capacity and the strong electron donating properties of the essential oil components and may partially explain the insecticidal activity found, with a recorded 70.70% repellency after 1 h of exposure to the essential oil, compared with the control. The essential oil exhibited notable in vitro anti‐lipoxygenase activity, with an IC_50_ value of 0.01 ± 0.002 μg mL^−1^ under the experimental conditions used. Moreover, molecular Docking outputs confirmed that the five main components of the essential oil bound well into the cavity of eukaryotic LOX‐5 enzyme. Additionally, SAF‐1 cell line cells were exposed to various concentrations of *M. marina* essential oil. The findings indicate that the viability of SAF‐1 cells was significantly affected only at a concentration of 5 mg mL^−1^. In conclusion, in addition to being a natural source of anti‐inflammatory chemicals for pharmaceutical uses, *Medicago marina* essential oil may also be an environmentally beneficial way to keep stored goods safe from bug infestations. The biological activities observed may reflect the combined effects of the major constituents of the essential oil, particularly in relation to its pronounced insecticidal, cytotoxic, antioxidant and anti‐lipoxygenase effects.

## Graphical abstract

Chemical characterization, in vitro biological activity and in silico analysis of essential oil from *Medicago marina* (sea medick) aerial parts.

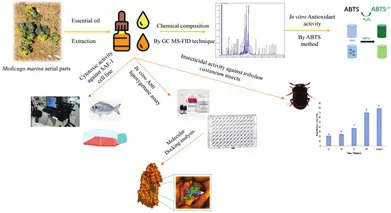

## 1 Introduction

The importance of herbal‐derived components in the health and disease management of animals, alongside their antioxidant properties, is garnering increasing research interest. Natural compounds exhibiting antioxidant activity have the potential to mitigate oxidative tissue damage by enhancing the protective capabilities of the endogenous antioxidant defense system (Schinella et al. 2002). Plants synthesize numerous secondary compounds with potent antioxidant properties. Previous studies have demonstrated that certain natural substances, including essential oils, may have biological, antioxidant, medicinal, and pharmacological applications (Anthony et al. 2012). Consequently, the screening of various plant species for natural antioxidants is a rapidly expanding field of research, driven by concerns regarding the adverse effects associated with synthetic antioxidants (Yang et al. 2010).

Essential oils (EOs) are naturally occurring compounds extracted from plants, exhibiting various biological properties such as insecticidal, antioxidant, and antibacterial effects, thereby serving as alternatives to synthetic substances (Mssillou et al. 2022). These oils are extensively utilized in the food industry as flavoring agents and antioxidants to preserve stored food crops, as well as in the medical, pharmaceutical, and cosmetic sectors, replacing synthetic chemicals that are considered cytotoxic (Frassinetti et al. 2011). The chemical constituents of EOs, characterized by conjugated carbon double bonds and hydroxyl groups, have the capacity to donate hydrogen, thereby mitigating oxidative stress and neutralizing free radicals (Diniz do Nascimento et al. 2020).

Recent findings indicate that vitamin C supplementation may attenuate the biological response to free radicals due to its pro‐oxidative properties. Alpha‐tocopherol can exhibit pro‐oxidant behavior at elevated concentrations, attributed to ROS interaction, and remains reactive without ascorbic acid. Although the presence and assimilation of antioxidants are crucial, there is a paucity of research on the implications of pro‐oxidants (Maliar et al. 2023).

In fact, research has shown that chain‐breaking phenolic antioxidants, such as Toc and Trolox, cannot neutralize the eugenol radical. ROS can interact with larger molecules, including cellular phospholipids, to generate alkyl‐ or phenoxy‐radicals (Fujisawa et al. 2002). Recently, EOs have been investigated as a pest management strategy, with several aromatic and medicinal plants demonstrating remarkable efficacy in pest control and grain stock preservation (de Araújo Ribeiro et al. 2020). This approach may help mitigate resistance resulting from the excessive use of synthetic pesticides, prompting research into natural alternatives that pose reduced risks to human health and the environment.

One of the main reasons for both quantitative and qualitative losses in food goods that are preserved is insect infestation. Food supplies may become contaminated by stored‐product insects during storage, transit, and commercialization, resulting in large financial losses and lower product quality (Hagstrum 2016). A number of pest species have chewing mouthparts that allow them to enter the protected food items and harm packing materials. Furthermore, infestation may take place prior to packaging, making it possible for insects or their stages of development to go unnoticed in the packaged goods (Marsin et al. 2020).

Essential oils have drawn a lot of attention for their pharmacological qualities, especially their anti‐inflammatory action, in addition to their possible application in preventing insect infestation of food items that are being preserved. Their usefulness as multipurpose natural products with uses ranging from food preservation to human health promotion is highlighted by their dual functioning. Consequently, this creates an urgent need for natural alternatives to address this issue. Repellents are substances that exert effects either locally or distally, hindering arthropods' ability to approach, land on, or bite human or animal integument (Katz et al. 2008). Research has demonstrated that essential oils, which are complex amalgamations of volatile compounds derived from various plant species, exhibit repellent properties against hematophagous arthropods, thus serving as a basis for commercial repellent formulations (Caballero‐Gallardo et al. 2011).

Conversely, inflammation is a significant health issue in animals. The human body contains lipoxygenases (LOXs), which play a critical role in initiating inflammatory responses. An excess of reactive oxygen species can provoke inflammation, leading to cytokine production and the activation of lipoxygenases. Cereals and legumes, particularly those from the Fabaceae family, are abundant sources of LOXs (Lampi et al. 2020). Given the adverse aspects of the LOX pathway, research on lipoxygenase inhibition is of considerable interest (Lončarić et al. 2021).

The utilization of antibiotics and chemotherapeutics as prophylactic interventions in intensive aquaculture has been extensively criticized due to their detrimental effects on both fish and the environment. These effects include immunosuppression, residue accumulation in tissues, and the facilitation of drug‐resistant pathogens (Elumalai et al. 2020). As a result, there is a growing emphasis on the exploration of alternative natural compounds (Reverter et al. 2014). Nature offers a vast array of biological and chemical diversity, and the unique structures of natural products present challenges for replication through chemical synthesis (Li et al. 2018). In recent years, there has been an increased interest in the study of medicinal plants, particularly for addressing diseases with significant societal impact, such as cancer (Ji et al. 2009).

Essential oils substances comprise secondary metabolites found throughout the plant or in specific parts such as fruits, seeds, flowers, roots, stems, and leaves (Jain et al. 2019). Fatty acids are essential for growth and health maintenance as they enhance the immune system and contribute to disease prevention (Morales et al. 2023). Numerous essential oils have exhibited cytotoxic properties (Loizzo et al. 2007), however, to the best of our knowledge, no previous studies have examined the cytotoxic activity of essential oils derived from *Medicago marina* .

The genus *Medicago* holds considerable significance, comprising 83 species with a broad geographical distribution (Flamini et al. 2003). Among these, the legume *M. marina* L., commonly referred to as sea medick, is distributed from Spain and northern Africa to mid‐Asia (Tava et al. 2020). This species prospers along coastal dunes and can be utilized for the stabilization and restoration of such ecosystems (Alías‐Villegas et al. 2015). Medicago species are recognized for various properties, including neuroprotective, antioxidant, immunomodulatory, and diuretic activities (Khairy et al. 2025), as well as, antimicrobial, antibiofilm, and anticoagulant effects (Basto‐Silva et al. 2020). Numerous in vivo studies have integrated plants, their extracts, or their essential oils into animal diets, particularly in fish feed (Han et al. 2017). However, to the best of our knowledge, there is a paucity of research on the effects of these plants, extracts, or EO in vitro (Beltrán et al. 2018).

The aim of the present work was to investigate several biological properties of this EO, including its antioxidant, anti‐lipoxygenase, and insecticidal activities. Additionally, molecular docking was performed for the five major components of the essential oil against LOX‐5 enzyme; finally, the cytotoxic effects of this oil on the SAF‐1 cell line have been likewise examined.

## 2 Material and Methods

### 2.1 Plant Material

*M. marina* aerial parts were collected in July during the blooming season on the southern Tunisian island of Djerba. The identification of the plant was determined by Prof. Fethia Skhiri, a botanist at the Higher Institute of Biotechnology of Monastir (ISBM), Tunisia. At the Faculty of Pharmacy in Monastir, Tunisia, a sample of the voucher was delivered to our laboratory (MM‐H).

The collect of *Medicago marina* plant material was done in accordance with institutional and municipal laws controlling the harvesting of wild plant species. In order to ensure that all biological material was acquired and used responsibly and sustainably, the study adheres to the principles of the Nagoya Protocol on Access to Genetic Resources and the Fair and Equitable Sharing of Benefits Arising from their Utilization.

### 2.2 Chemical Composition of the *M. marina* Essential Oil

The essential oil of *M. marina* was obtained through hydrodistillation (Clevenger) for 4 h and subsequently analyzed for its chemical composition using GC‐MD‐FID analysis with an HP‐5 capillary column (30 m × 0.25 mm, 0.25 μm film thickness) (Melliti et al. 2024). The sequence of n‐hydrocarbons was compared with the linear retention indices. The constituents were identified through computer‐assisted matching against the commercial Agilent (PBM) search format, and the retention times were calculated utilizing pure authentic samples, where available. The mass spectral library for NIST 2020 was employed (Kilani et al. 2005).

### 2.3 Antioxidant Effect of the Essential Oil

The 2,2′‐azino‐bis‐3‐(ethylbenzothiazoline‐6‐sulphonic acid) (ABTS) method was employed to evaluate the antioxidant capacity of the EO (Arnao et al. 2001). This method is predicated on the ability of the sample's antioxidants to reduce the radical cation of ABTS, as evidenced by the decolorization of ABTS· + and the subsequent measurement of the quenching of absorbance at 730 nm. This activity, expressed as ascorbic acid equivalents (mmol) mg, is calculated by comparing the sample's results with an ascorbic acid standard curve. In accordance with the experimental protocol, 950 μL of the cation ABTS· + was combined with 25 μL samples of the EO at concentrations of 0.625, 1.25, 2.5 and 5 mg mL^−1^, which had been previously adjusted using phosphate‐buffered saline (PBS). The subsequent decrease in absorbance was measured using a spectrophotometer (BOECO S‐22 UV/Vis), with PBS serving as the reaction blank. Each sample was analyzed in triplicate to ensure accuracy and reliability of the results.

### 2.4 Insecticidal Activity of the Essential Oil

#### 2.4.1 Repellent Activity Test

The repellent effect of *M. marina* EO on *Tribolium castaneum* was examined using the preferential zone method on filter paper (L. L. McDonald et al. 1970). Filter paper discs, each with a diameter of 8 cm, were divided into two equal halves (4 cm each). A 100 μL aliquot of the serial dilution of the EO (from 2 to 0.5 mg/mL), was applied to one half of the filter paper, while the other half received 100 μL of acetone, serving as a solvent control. Five replicates were conducted for each extract. Following a 15‐min interval to allow for complete solvent evaporation, the two halves of the discs were rejoined using adhesive tape. These reconstituted paper discs were then placed in Petri dishes, with 20 adult insects positioned at the center of each disc. The mean percentage of repulsion calculated at each time point was categorized into one of the different classes ranging from 0 to V (S. McDonald 1981). The repellency rate (PR) was calculated using the formula: PR (%) = [(Nac − Neo), (Nac − Neo)] × 100, where Nac represents the number of individuals present in the acetone‐treated section, and Neo denotes the number of individuals present in the section treated with EO diluted in hexane.

#### 2.4.2 Fumigation Activity Test

The impact of essential oil (EO) on the inhalation mortality rate of insects was investigated. In 40 mL containers, 10 insects were introduced, and filter paper discs moistened with varying doses of EO were affixed to the container caps. Four doses were evaluated (1, 2, 4, and 8 μL), corresponding to concentrations of 25, 50, 100, and 200 μL L^−1^ air. For each concentration, five replicates were conducted in each container. Control containers, devoid of EO, were prepared for each test, each containing 10 individuals. Mortality was determined by counting the number of deceased insects at 24 h and 48 h post‐experiment initiation. An insect was deemed deceased if it exhibited no movement of its antennae or legs (Hamouda et al. 2014). Mortality was calculated and adjusted for natural mortality (Mt) observed in the control using Abbott's formula (Mc = Mo − Mt)/(100 − Mt × 100), where Mc represents the corrected mortality rate, Mo denotes the mortality rate in treated containers, and Mt. indicates the observed mortality rate in the control.

### 2.5 Cytotoxic Activity of the EO on SAF‐1 Cells

The SAF‐1 cell line (RRID: CVCL_4295) (ECACC n° 00122301) was cultured in 25 cm^2^ plastic tissue culture flasks (Nunc). The growth medium employed was L‐15 Leibowitz medium (Life Technologies), supplemented with 10% fetal bovine serum (FBS, Life Technologies), 2 mM L‐glutamine (Life Technologies), and 100 i.u. mL^−1^ penicillin (Life Technologies). The cells were maintained at 25°C in an environment with 85% humidity. Standard trypsinization techniques were utilized to harvest cells in the exponential growth phase, involving a brief treatment with 0.25% trypsin in PBS s (pH 7.2–7.4), followed by centrifugation at 1000 rpm for 5 min at 25°C to collect the detached cells. Cell viability was assessed using the trypan blue exclusion test (Fazio et al. 2017). For cytotoxicity assays, SAF‐1 cells were harvested at approximately 80% confluency and seeded into 96‐well tissue culture plates at a density of 50,000 cells per well in 100 μL portions. After a 24‐h incubation at 25°C, the growth medium was replaced with 100 μL per well of the test extracts at appropriate dilutions. The EO concentrations ranged from 5 to 0.005 mg mL^−1^. The cells were then incubated for an additional 24 h. Control samples received an equal volume. To evaluate cell viability, the MTT assay was conducted after a 24‐h incubation period at 25°C. This method is based on the conversion of the yellow, water‐soluble tetrazolium salt (3‐(4,5‐dimethylthiazol‐2‐yl)‐2,5‐diphenyltetrazolium bromide) (MTT, Sigma) into a blue, water‐insoluble formazan compound by mitochondrial succinate dehydrogenase. Following incubation with leaf extracts, SAF‐1 cells were rinsed with PBS and exposed to 200 μL per well of MTT (1 mg mL^−1^). After an additional 4 h of incubation, the cells were washed, and the formazan crystals were dissolved using 100 μL of DMSO per well. The plates were then shaken in the dark at 100 rpm for 5 min, and absorbance was measured at 570 and 690 nm with a microplate reader (Berridge and Tan 1993).

### 2.6 Anti‐Lipoxygenase Activity of the EO

The anti‐lipoxygenase assay was performed using linoleic acid as the substrate and the lipoxygenase enzyme (Eshwarappa et al. 2016). The plant EO sample, at concentrations ranging from 0.0025 to 0.08 μg mL^−1^, was dissolved in 0.25 mL of 2 M borate buffer. To achieve a final concentration of 20,000 U mL^−1^, 0.25 mL of soybean lipoxygenase enzyme solution at pH 9.0 was added. The mixture was incubated for 5 min at 25°C. Subsequently, 1.0 mL of 0.6 mM linoleic acid solution was added and thoroughly mixed, with absorbance measured at 234 nm. Dexamethasone was used as the reference standard. The percentage of inhibition was calculated by comparing the absorbance of the control with that of the test sample. A dose–response curve was employed to determine the IC values, where IC_50_ represents the concentration required to achieve 50% of maximal scavenging capability. All tests and analyses were conducted in duplicate.

### 2.7 Molecular Docking Procedure

Molecular docking studies were performed by using Auto Dock 4.2 program package (Trott and Olson 2010). The X‐ray crystallographic structure S663D Stable‐5‐LOX in complex with Arachidonic Acid (PDB: 3v99) was obtained from the RSCB protein data bank (Gilbert et al. 2012). The optimization of all the geometries of compounds was carried out using ACD (3D viewer) software (http://www.filefacts.com/acd3d‐viewer‐freeware‐info). For the receptor preparation, the water molecules (H_2_O) were removed, and the missing hydrogens and Gasteiger charges were added to the system during the preparation of the receptor input file (PDBQT). The visualization and analysis of interactions were performed using Discovery Studio 2017R2 (https://www.3dsbiovia.com/products/collaborative‐science/biovia‐discovery‐studio/) and PyMOL 0.99rc6 (Schrodinger 2015).

### 2.8 Statistical Analyses

Statistical differences among the groups were evaluated using one‐way ANOVA, followed by Tukey's multiple comparisons test. The normality of the variables was verified through the Shapiro–Wilk test, and homogeneity of variance was assessed using the Levene test. A significance level of 95% was maintained in all analyses (*p* < 0.05). No chi‐square or correlation analyses were performed in this study. All data were processed using the SPSS for Windows software application (version 25.0, SPSS Inc., Chicago, USA).

## 3 Results and Discussion

This study aimed to investigate the various biological characteristics of the EO of sea medick, including antioxidant capacity (the ability to protect cells from free radical damage), anti‐lipoxygenase potential (the capacity to reduce inflammation by inhibiting a specific enzyme), and insecticidal efficacy (the ability to eliminate or deter insects). The objective was to examine these diverse biological effects to identify potential new applications for the EO.

### 3.1 Chemical Composition of *M.Marina* Essential Oil

**FIGURE 1:**
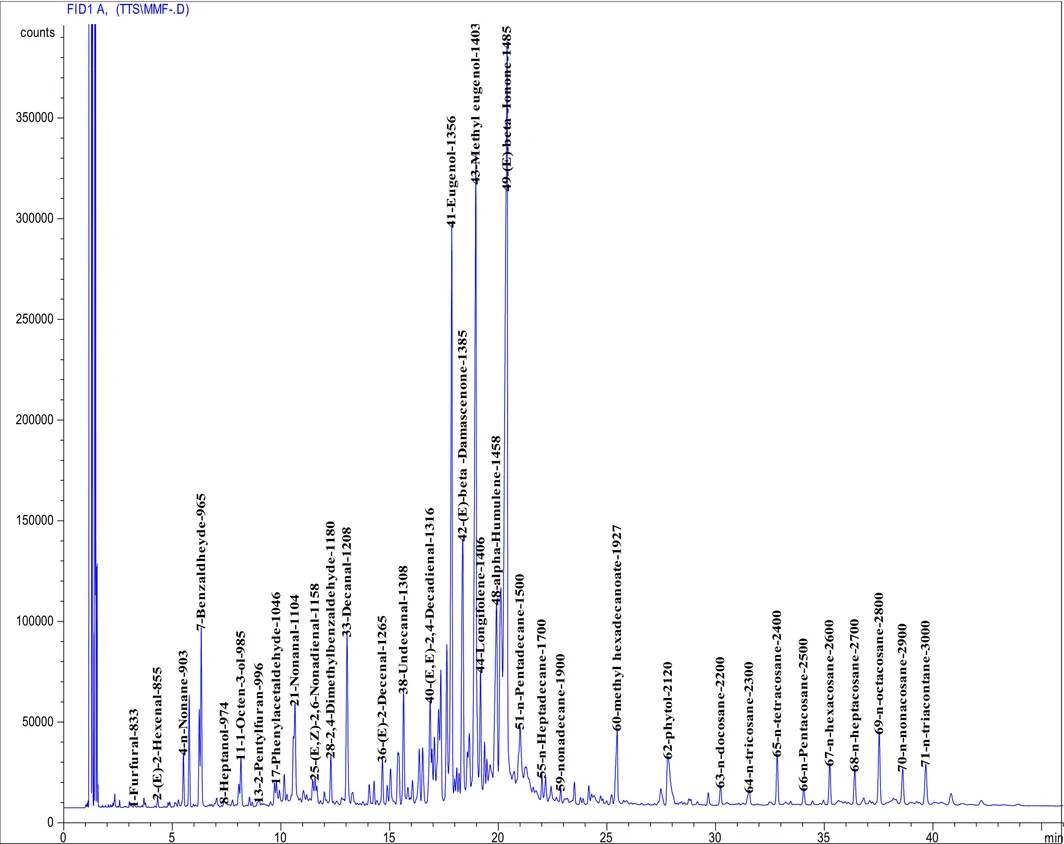
GC‐FID chromatogram of *M. marina* essential oil.

As previously reported (Melliti et al. 2025), phytochemicals in sea medick essential oil were investigated and the findings announced that this oil was rich in monoterpenes including β‐ionone and sesquiterpenes such as α‐humulene. Phenols were also abundant in this oil, such as eugenol and methyl eugenol, as well as carotenoid derivatives like (E)‐β‐damascenone (Table 1). Nonetheless, β‐ionone (20.48%), methyl eugenol (12.46%), and eugenol (8.79%) were the primary constituents of Tunisian *M. marina* essential oil (Figure 1) (Flamini et al. 2003). Numerous studies have demonstrated that a number of factors, including the species to which the plant belongs, the plant material used (leaves, flowers, twigs, fruits, etc.), climate types, geographical location, and harvest time, can affect the chemical composition of essential oils (Gobbo‐Neto and Lopes 2007).

**TABLE 1:** Major compound classes of *M. marina* essential oil.

Chemical compounds	Abundance (%)
(E)‐β‐ionone	17.677
Methyl eugenol	10.759
Eugenol	8.86
(E)‐β‐damascenone	4.337
α‐Humulene	4.323

Chemical classes	Relative abundance (%)
Ketone monoterpenes	23.224
Phenols	21.377
Aldehydes	13.702
Hydrocarbons	9.925
Sesquiterpenes	9.515
Esters	2.829
Ketones	1.862
Hydrogenated diterpenes	1.701
Alcohols	0.996
Oxygenated monoterpenes	0.825
Other	0.788
Total identified (%)	87.607

### 3.2 Antioxidant Activity of *M. marina* EO

The total antioxidant capacity was assessed by ABTS assay. Ascorbic acid equivalents (AAE) values were determined using a calibration curve of standard ascorbic acid.

Results showed considerable inhibitory potential of *M. marina* EO with an AAE value of approximately 3.61 mg/mL ±0.4 mg/mL at a concentration of 5 mg/mL, and an IC50 value of 1.7 ± 0.4 mg mL^−1^ (Table 2).

**TABLE 2:** ABTS radical scavenging activity of *M. marina* essential oil compared with Vitamin C.

Concentration (mg/mL)	Vitamin C (% inhibition)	Eo (% inhibition)	AAE (mg/mL eq Vit C)
0.625	25.3 ± 0.66^f^	18.7 ± 0.7^a^	0.25 ± 0.34^a^
1.25	50.1 ± 0.8^d^	36.5 ± 0.5^b^	0.51 ± 0.55^a^
2.5	62.2 ± 0.7^c^	68.2 ± 0.3^c^	2.16 ± 0.3^b^
5	98.1 ± 0.4^a^	81.3 ± 0.2^d^	3.61 ± 0.4^c^
IC50 (mg/mL)	1.24 ± 0.33^A^	1.7 ± 0.4^B^	

*Note:* Results are expressed as mean standard deviation (SD) of three independent experiments (*n* = 3). Different letters indicate significant differences between treatment groups (*p* < 0.05).

A s shown in Table 2, *M.marina* essential oil demonstrated a considerable significant and dose dependent antioxidant effect (*p* < 0.05).

The IC 50 value of 1.7 ± 0.4 mg/mL was low. Despite being statistically different (*p* < 0.05, Group B) from the pure Vitamin C standard (IC 50 = 1.24 ± 0.33 mg/mL, Group A), this small difference clearly highlights the raw essential oil matrix's effective intrinsic hydrogen‐donating or electron‐transfer capabilities.

The antioxidant properties of volatile oils are determined not by individual components but by the interaction of all constituents within the essential oil, due to their complex chemical composition containing molecules with various functional groups. The interaction among components can diminish antioxidant activity (antagonistic effect) or enhance it (synergistic effect) (Andrade et al. 2013). Additionally, EO from a Lamiaceae species (*Thymus pectinatus)* such as α‐ and γ‐terpinene, limonene, p‐cymene, linalool, and others may exhibit these antagonistic or synergistic effects (Vardar‐Ünlü et al. 2003).

EO from *O. sanctum* , containing eugenol, exhibited limited antioxidant effects. The absence of antioxidant activity in methyl eugenol found in *O. gratissimum* and *O. sanctum* EO may be due to the lack of proton(s) or electron delocalization groups (Joshi 2013).

Other studies report that croton EOs with higher eugenol content show enhanced capacity to stabilize free radicals (Oliveira et al. 2021). EOs with methyl eugenol and eugenol as primary constituents have been linked to strong antioxidant activity (Alminderej et al. 2020). Antioxidants mitigate harmful free radicals, which can cause oxidative stress and chronic conditions (Chandimali et al. 2025).

EOs with antioxidant activity may offer cellular protection, reducing risk of cardiovascular diseases, cancer, and neurodegenerative disorders (Abd Rashed et al. 2021). Antioxidant‐rich EOs can be used in food preservation to inhibit lipid oxidation and extend shelf life, and enhance antioxidant capacity of fermented foods (Petcu et al. 2023). These EOs have applications in cosmetics and skincare, where their properties may help reduce aging signs and protect skin from environmental stressors (Javed et al. 2024). This interest has spurred investigation of plant‐derived sources, as shown in this study. As research progresses, antioxidant applications continue to expand across fields.

### 3.3 Insecticidal Effect of *M. marina* EO

#### 3.3.1 Repellency Effect of the Essential Oil

**FIGURE 2:**
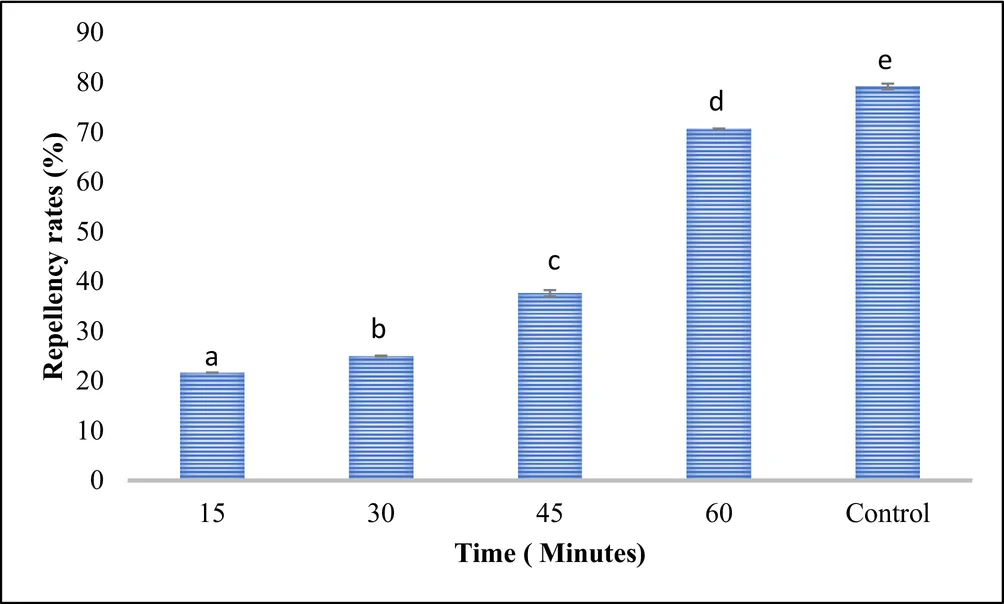
The repellency test results of *M. marina* essential oil at a concentration of 2 mg/mL as compared to the control were assessed at intervals of 15, 30, 45, and 60 min following exposure to *Tribolium castaneum* insects. The values (*n* = 5) are means ± standard deviations (SD). Means with the same letter do not differ substantially at *p* < 0.05.

To the best of our knowledge, there is no existing documentation regarding the insecticidal activity of *M. marina* EO. In this study, we evaluated its repellent effects against *T. castaneum* adults. The repellency of *M. marina* essential oil was assessed at a single concentration (2 mg/mL) to get an initial evaluation of its behavioral influence on insects. There was no discernible dose‐dependent effect under these experimental circumstances, and only the highest concentration (2 mg/mL) was tested due to sample availability. Our findings indicated an interesting repellent activity (PR = 70.7% ±1 (Class IV)) after 1 h of exposure to *M. marina* EO only at the concentration of 2 mg/mL, in comparison to the control (PR = 79.16%, *p* < 0.05) (Figure 2). For instance, the EO of *T. foenum‐graecum* , a member of the Fabaceae family, exhibits a weaker repellent effect (33.70%) (Class II) (Naimi et al. 2022).

Furthermore, it is noteworthy that when examined individually, *Phytolacca Dodecandra* EO constituents have shown enhanced repellent properties against *T. castaneum* compared with both EOs and commercial repellents (Wang et al. 2008). A recent investigation demonstrated a correlation between insecticidal activity and the phytochemicals present in 20 American native plants EO (Duque et al. 2023). Additionally, other studies conducted on three essential oils from *Artemissia herba*, *origanum vulgare* , and *ruta montana* have suggested that specific active components in the signal's composition elicit preferential reactions from insects exposed to chemical signals, such as EOs (Bouzeraa et al. 2019).

Research indicates that plant EOs ( *Croton grewioides* , cinnamon oil, horseradish oil, mustard oil) and their metabolites possess insect‐repelling properties (Kim et al. 2003; Papachristos and Stamopoulos 2002). These repellent characteristics could be primarily associated with monoterpenoids and sesquiterpenes (Kumar et al. 2022). The majority of insect‐repellent compounds are oxygenated, featuring a hydroxyl group attached to a primary, secondary, or aromatic carbon. Certain metabolites, such as linalool, α‐terpineol, and limonene, which have a hydroxyl group linked to a tertiary carbon, demonstrate reduced activity. This suggests that the type of carbon to which the hydroxyl group is attached may affect repellency (Mudrončeková et al. 2019). Volatility is a critical factor in the effectiveness of repellents (Xu and Turlings 2018). The deterrent properties of basil essential oil are attributed to monoterpenes and sesquiterpenes; however, their efficacy is influenced by variables such as insect type, plant species, and exposure duration, all of which impact their overall effectiveness (Toudert‐Taleb et al. 2021). The biological actions of β‐ionone include both insect attraction and repellent properties, and experiments on repellency have shown significant activity of R‐(+)‐limonene (Malacrinò et al. 2016; Paparella et al. 2021). In the case of eugenol, it exhibited strong repellent effects against four beetle species, achieving an overall repellency rate of 80%–100% (Obeng‐Ofori and Reichmuth 1997).

Moreover, it has been extensively documented that phenolic chemicals included in essential oils play a major role in their insecticidal and repellency properties. Their capacity to disrupt respiratory enzymes, cuticular integrity, and insect neurotransmission is the primary cause of their bioactivity. Particularly, phenols like eugenol, thymol, and carvacrol have shown potent poisonous and repellency effects against a variety of agricultural pests, particularly insects that feed on stored products. These substances can have a variety of effects, such as blocking octopamine receptors, interfering with GABA‐gated chloride channels, and causing oxidative stress in insect cells, which eventually results in behavioral avoidance and death. Therefore, increased insecticidal and repellent performance is frequently associated with the presence of phenolic components in essential oils (Isman 2006; Nerio et al. 2009; Pavela 2015).

In conclusion, the chemical composition of the essential oil can be taken into consideration in relation to the insecticidal action shown in this investigation. Numerous components included in the oil may interact with various physiological or neurological targets to produce insecticidal effects. However, as interactions between constituents may affect the total potency, the activity of the entire essential oil cannot always be anticipated from the abundance of a single ingredient. To ascertain each component's contribution to the observed insecticidal action, more research utilizing individual components and specific combinations would be necessary.

#### 3.3.2 Fumigation Activity

Under the evaluated experimental conditions, *Tribolium castaneum* exposed to *M. marina* essential oil did not exhibit any mortality. As a result, at the concentrations tested, the essential oil showed no discernible insecticidal efficacy against this species.

In fact, bioactive compounds might not even kill insects directly, as in earlier research, but they might cause a dramatic decrease in oviposition and a variety of developmental abnormalities that could totally stop an individual's development (Aimad et al. 2021). A recent study demonstrated that a performed mortality test against *E. eugeniae* and *A. salina* revealed no dead earthworms after seven days of treatment with the seed‐derived essential oil of *Acacia nilotica* , which belongs to the *Fabaceae* family (Perumal et al. 2023). Further investigations should be conducted, including oviposition inhibition assays, developmental impact studies, or long‐term exposure experiments to evaluate sublethal effects of *M. marina* EO on *T. castaneum* populations.

### 3.4 Cytotoxic Activity of *M. marina* EO on SAF‐1 Cells

**FIGURE 3:**
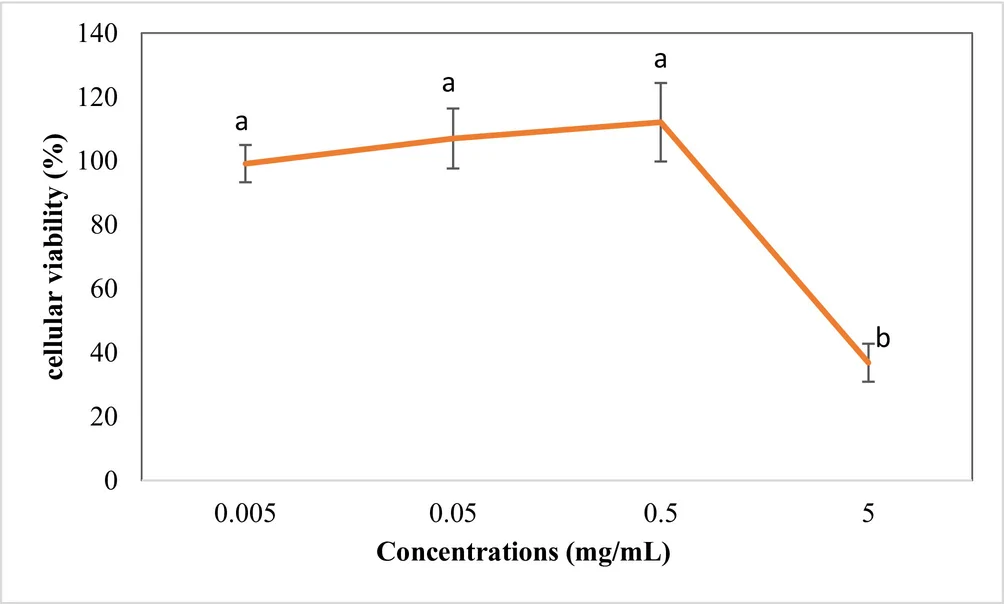
Viability (expressed as percentage) of SAF‐1 cell line after 24 h of incubation with *M. marina* essential oil. When compared to untreated cells, cytotoxicity data are expressed as a percentage of viability. The findings, which are shown as mean ± SD, are indicative of a minimum of three separate trials. Significant differences between treatment groups (*p* < 0.05) are indicated by different letters.

The potential cytotoxic effects of the EOs were assessed at several concentrations ranging from 0.005 to 5 mg mL^−1^ on SAF‐1 cells (Figure 3). The results demonstrated that there was no statistically significant impact on the viability of the cells when the EO was used at lower concentrations than 5 mg mL^−1^. However, at the highest concentration (5 mg mL^−1^), *M. marina* EO caused the mortality of 63.2% of the cells. Our findings also showed that EC50 = 4.088 mg mL^−1^ (Figure 3). As already mentioned, a plant's biological activity is associated with the totality of chemical substances present within it (Riaz et al. 2023).

Monoterpenes are small, lipophilic molecules that readily penetrate a biomembrane's lipid bilayer, accumulating internally and enhancing its fluidity. Phenols can dissociate to generate a phenolate ion, with the polar portion aggregating along the membrane's periphery (Herrmann and Wink 2011). Multiple chemical constituents frequently contribute to synergistic cytotoxic activity (Melo et al. 2014).

High concentrations of eugenol exhibit cytotoxicity, and tissue damage may result from direct contact between essential tissue and eugenol‐containing materials (Escobar‐García et al. 2016). Methyl‐eugenol, at concentrations ranging from 10 to 500 mM, induced unscheduled DNA synthesis (UDS) in rodents (Burkey et al. 2000). Eugenol demonstrates various forms of toxicity, including direct tissue damage, dermatitis, allergic reactions, liver dysfunction, disseminated intravascular coagulation, severe hypoglycemia, and even mortality from multiple organ failure, observed both in vivo and in vitro. Pure eugenol inhibits cell migration and alters prostaglandin synthesis at concentrations exceeding 10^−4^ mol L^−1^, affecting mitochondrial activity, cellular respiration, and cell membrane enzymatic activity (Liang et al. 2015). Mouse fibroblasts demonstrated resistance to cell death when exposed to 10‐3 M eugenol for durations up to 12 h (Hume 1984).

*M. marina* EO was found to be rich in β‐Ionone compound. β‐Ionone derived chalcones exhibit cytotoxic effects against cancerous cells, inducing apoptotic signs and suppressing cell growth by arresting the cell at the G0 phase (Sharma et al. 2013). Incubation of K562 cells with 200 μM beta‐ionone resulted in an increased apoptotic rate (Faezizadeh et al. 2016).

Studies evaluated the cytotoxic effect of candle bush ( *Senna alata* ) against breast cancer (MCF‐7) and normal human mammary epithelial cells (MCF10A). Two extracts demonstrated no toxicity to brine shrimp: the n‐hexane fraction and 8 0% ethanolic crude extract. Notably, MCF10A did not exhibit adverse effects from any of the extracts (Chahardehi et al. 2021).

Recent results from the cytotoxicity assay performed on the SAF‐1 cell line, following exposure to *M. marina* essential oil (EO), reveal that within the limited concentration range tested, there are no significant effects on cell viability. This observation implies the absence of harmful compounds in these fractions. In light of previous reviews, it is recommended that eugenol formulations be employed with careful consideration of the risk/benefit ratio, akin to other pharmacological agents, due to their known cytotoxic properties. Proper application should take into account the method and site of administration, dosage, the condition of the tissue to be treated, and the duration of exposure to the substance (Escobar‐García et al. 2016).

To conclude, the cytotoxicity findings offer a crucial complement to the essential oil's insecticidal and antioxidant properties. The discovered effects on fish fibroblast cells provide information on the biological safety profile and potential impacts on non‐target cells, whilst the latter activities may suggest possible functional or pesticidal applications. As a result, both the essential oil's intended actions and any potential toxicity should be taken into account when assessing its biological potential. Specifically, comparing the concentrations causing insecticidal effects with those linked to cytotoxicity may offer initial insights into the oil's selectivity. To determine its safety and environmental compatibility, more research utilizing different non‐target cell types and in vivo models is required. However, such comparisons should be read cautiously.

### 3.5 Anti‐Lipoxygenase Inhibition Test

The EO of *M.marina* demonstrated statistically considerable anti‐lipoxygenase activity (IC50 = 0.01 μg mL^−1^) in comparison to dexamethasone, which served as the control (IC50 = 0.005 μg mL^−1^). The tested essential oil induced 75.21% of enzyme inhibition at 0.08 μg/mL and this activity was significant and dose dependent (*p* < 0.05) (Table 3). Using the same experimental assay and computation method as the reference chemical dexamethasone, the anti‐lipoxygenase activity of the essential oil was assessed. With an IC50 value of 0.01 μg mL^−1^ for the essential oil and 0.005 μg mL^−1^ for dexamethasone under the identical experimental conditions, the essential oil exhibited pronounced in vitro anti‐lipoxygenase activity, although its inhibitory potency was lower than that of dexamethasone. The composition and proportions of volatile oil components in essential oils may influence their biological activities, including antioxidant and anti‐inflammatory properties (Dhifi et al. 2016).

**TABLE 3:** Results of the anti‐lipoxygenase activity test expressed as IC50 values.

Concentrations (μg/mL)	Inhibition percentages (%)	
	MM EO	Dexamethasone
0.0025	18.6 ± 0.3^a^	21.4 ± 0.22^a^
0.005	30.21 ± 0.2^b^	50.06 ± 0.1^b^
0.01	50.12 ± 0.28^c^	51.04 ± 0.28^b^
0.02	58.91 ± 0.19^d^	61.72 ± 0.17^c^
0.04	66.45 ± 0.1^e^	66.51 ± 0.11^d^
0.08	75.21 ± 0.37^f^	90.21 ± 0.9^e^
IC50 (μg/mL)	0.01 ± 0.002^A^	0.005 ± 0.001^B^

*Note:* The results, displayed as mean ± SD, are representative of at least three different trials. Different letters indicate significant differences between treatment groups (*p* < 0.05).

Enzyme kinetic experiments revealed that eugenol inhibits lipid peroxidation through a non‐competitive mechanism, altering the maximum velocity while leaving the Michaelis–Menten constant unchanged (IC50 = 380 μM). This inhibitory mechanism suggests that eugenol interacts with fatty acid radical intermediates via its hydroxyl radical scavenging properties, thereby mitigating the progression of lipid peroxidation rather than directly inactivating the enzyme (Naidu 1995).

Other studies have indicated that eugenol inhibits 5‐lipoxygenase (5‐LO) through a non‐competitive mechanism (Raghavenra et al. 2006). Additionally, eugenol has been reported to exhibit COX‐2 inhibitory action in LPS‐stimulated murine macrophages in vitro (Irie 2006; Mahapatra et al. 2009). Previous research has demonstrated that the anti‐inflammatory properties of eugenol may be attributed to its ability to inhibit prostaglandin production or the release of other endogenous mediators.

Moreover, it has been extensively documented that phenolic compounds found in essential oils have strong anti‐inflammatory qualities, especially when it comes to the modulation of important enzymes in the arachidonic acid pathway. One of the most significant of these mechanisms is the inhibition of 5‐lipoxygenase (5‐LOX), which reduces the production of pro‐inflammatory leukotrienes. Eugenol, thymol, and other phenylpropanoid derivatives are examples of natural phenols that have been shown to inhibit lipid peroxidation and disrupt inflammatory enzymatic oxidation processes. These substances have been demonstrated to affect several inflammatory signaling pathways, including COX‐2 expression and NF‐κB activation, in addition to their anti‐lipoxygenase activity, underscoring their pleiotropic anti‐inflammatory potential (Bakkali et al. 2008; Baylac and Racine 2003; Miguel 2010).

The essential oil's anti‐lipoxygenase activity offers crucial proof of its biological potential. Under the experimental conditions, the essential oil showed an IC50 of 0.005 μg mL^−1^, indicating significant inhibitory activity at a low dose. According to earlier research, lipoxygenase activity can be interfered with by a number of terpenoid components that are frequently present in essential oils. This suggests that the observed inhibition may be related to the combined effect of the oil's main constituents (Naidu 1995). Furthermore, although the activity of the entire oil cannot be assigned to a single chemical based only on its abundance, interactions between the essential oil constituents and the enzyme may contribute to the observed inhibitory impact. However, the current results show inhibition in vitro and should not be taken as direct proof of anti‐inflammatory effectiveness in vivo. To validate the biological significance of this activity, more mechanistic, cellular, and in vivo research is needed.

Although in vivo validation is still an active area of research, several reviews have verified that plant‐derived polyphenols function as potent 5‐LOX inhibitors, supporting their potential as natural anti‐inflammatory medicines. These results are corroborated by experimental research on essential oils, which shows that phenolic‐rich extracts can directly decrease 5‐LOX activity and lower the synthesis of inflammatory mediators, contributing to their overall pharmacological benefits (Fraga et al. 2019; Werz 2002).

Our results provide preliminary evidence of biological activity and further investigations are essential to improve the robustness.

### 3.6 Molecular Docking Analysis

Drug discovery is a complex, multifaceted endeavor in which selecting the appropriate lead compound is indispensable to a project's success. For years, molecular docking has been widely used as a key computational technique in structure‐based drug design. In this context, this modeling method was utilized in the present study to propose a potential mechanism of action consistent with the recorded anti‐inflammatory potential by using 5‐lipoxygenase target receptor (pdb: 3v99) and the main phytoconstituents. Generally, the binding site of eukaryotic LOXs is highly conserved, motivating us to use LOX‐5 as a structural model to characterize the interactions involved. As known, the binding site of LOX‐5 is primarily composed (roughly 50%) of hydrophobic amino acids (Trp147, Phe169, Phe177, Leu179, Phe229, Leu368, Ile406, Ala410, Phe555, Ala603, Leu607, Phe610, Val671, and Ala672) (Zeraik et al. 2021).

The results shown in Table 4 demonstrate that several ligands showed appreciable binding affinities, ranging from −4.2 to −5.7 kcal/mol, even though none of the docked phytomolecules achieved a docking score significantly better than that of the dexamethasone standard.

**TABLE 4:** Binding energy of the docked compounds in the binding cavity of ‘lipoxygenase’ (PDB: 3v99).

Docked compounds	Interacting residues	Binding energy (kcal/mol)
Eugenol 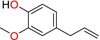	van der Waals: Lys409; H‐bond: Ala410, Gln413; Pi‐Sigma: Ala410; Pi‐Pi Stacked: Phe177; Alkyl/Pi–Alkyl: Phe177, Leu607	−4.5
(E)‐β‐damascenone 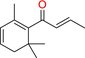	van der Waals: His367, Leu368, Ile406, Gln413, Leu607; Pi‐Sigma: His372; Alkyl/Pi–Alkyl: Phe177, Ala410	−5.2
Methyl eugenol 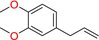	van der Waals: Ile406, Lys409; H‐bond: Phe177; Carbon Hydrogen Bond: Ala410, Gln413; Pi‐Sigma: Ala410; Alkyl/Pi–Alkyl: Leu368, His372, Ala410	−4.2
α‐humulene 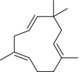	van der Waals: Gln413; Alkyl/Pi–Alkyl: Phe177, His372, Ile406, Lys409, Ala410, Leu607	−5.7
(E) β‐ionone 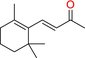	van der Waals: Leu368, Lys409, Gln413; Carbon Hydrogen Bond: His367; Pi‐Sigma: His372; Alkyl/Pi–Alkyl: Phe177, Ile406, Ala410	−5.7
Dexamethasone (standard) 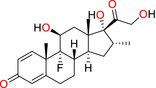	van der Waals: Phe177, Leu179, Asn180, His367, Asn554, Gln557, Gln609, Phe610, Ala672; H bond: His372, His550; Carbon Hydrogen Bond: Lys183, Leu607, Val671; Alkyl/Pi–Alkyl: Leu607	−7.4

According to the findings, α‐humulene, (E)‐β‐ionone, and (E)‐β damascenone exhibited moderate binding affinities (−5.7 and −5.4 Kcal/mol respectively) toward the target protein, whereas methyl eugenol and eugenol showed weak binding affinities (−4.2 and −4.5 Kcal/mol respectively) in comparison with dexamethasone.

These findings do not support biological activity, but they do point to possible interactions between the discovered phytochemicals and the active site of the enzyme.

As a result, the biological effects seen in vitro could be the consequence of several components working through additive rather than synergistic pathways.

In fact, recent studies have shown that a combined effect of several natural constituents can produce a more effective overall binding than one of the ligands alone (Rigby 2024).

**FIGURE 4:**
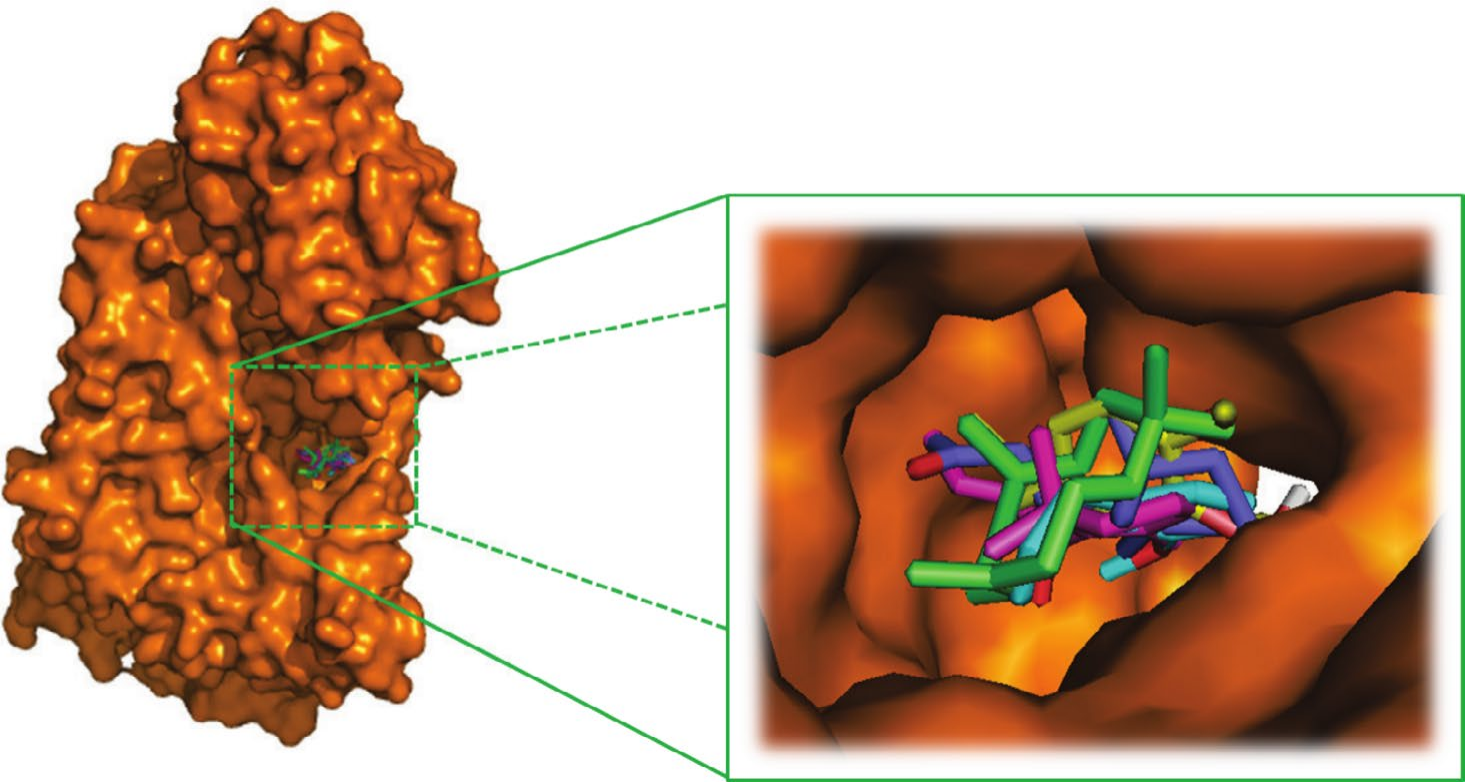
The 3D binding modes of the docked ligands (a) Eugenol (cyan color), (b) (E)‐β‐damascenone (purple color), (c) Methyl eugenol (yellow color), (d) α‐humulene (green color), and (e) (E)β‐ionone (blue color) in the binding cavity of ‘lipoxygenase’ (pdb: 3v99).

**FIGURE 5:**
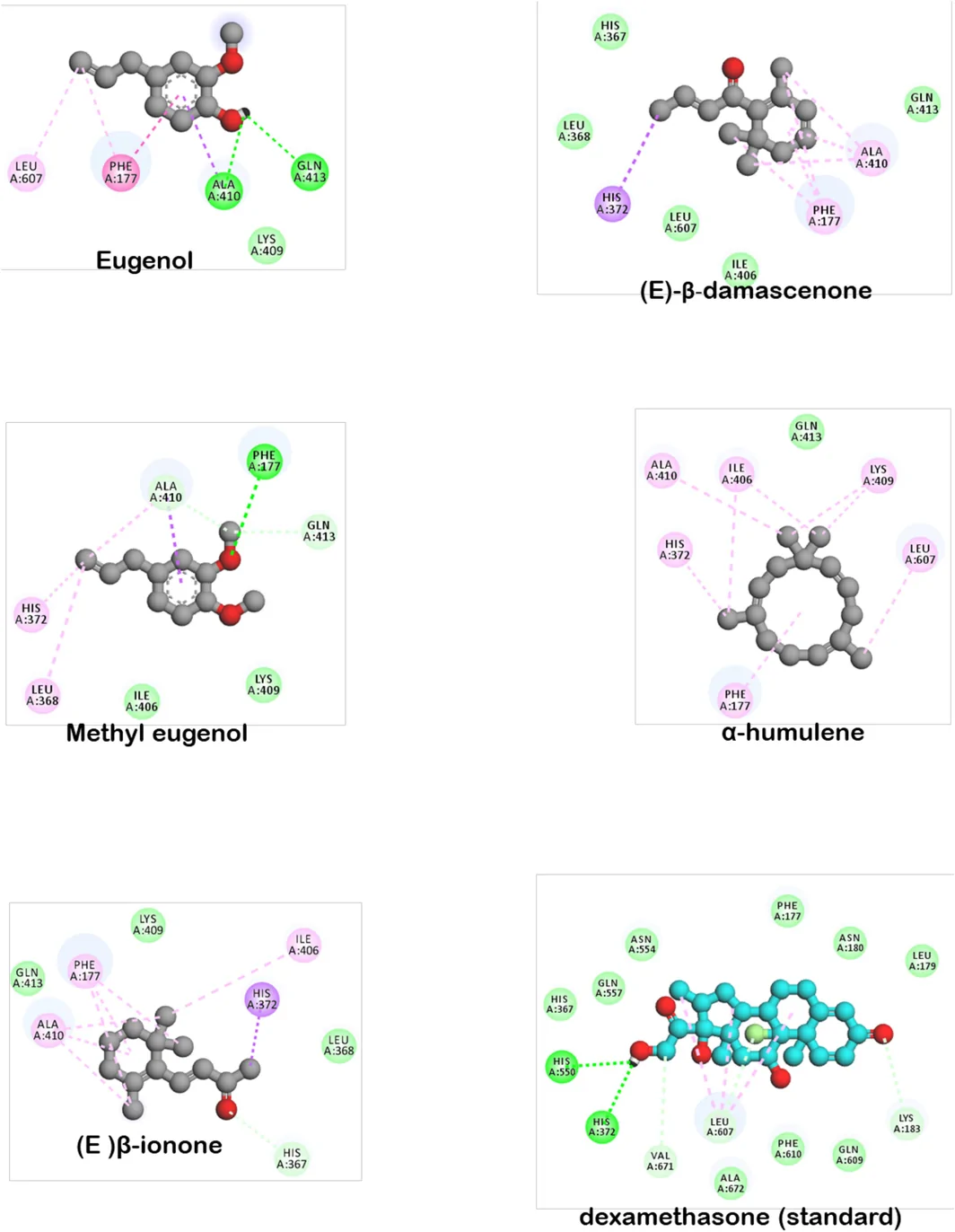
The 2D binding modes of the docked ligands (a) Eugenol, (b) (E)‐β‐damascenone, (c) Methyl eugenol, (d) α‐humulene, and (e) (E) β‐ionone in the binding cavity of lipoxygenase (pdb: 3v99).

The 3D model in Figure 4 shows that the docked phyto‐ligands fit well in the LOX‐5's binding cavity. For more details, it can be observed from the tabulated data that the most bioactive compounds are ‘α‐humulene’ and ‘(E) β‐ionone’ which exhibited the best docking score (binding energy value = −5.7 kcal/mol) compared with their analogues. Indeed, as Figure 5 shows, ‘α‐humulene’ exhibited several hydrophobic intermolecular interactions as Alkyl/Pi–Alkyls with the amino acid sequence: Phe177, His372, Ile406, Lys409, Ala410 and Leu607 while ‘(E)β‐ionone’ is involved in Carbon Hydrogen Bond with His367, Pi‐Sigma with His372 as well as Alkyl/Pi–Alkyl contacts with Phe177, Ile406 and Ala410. Furthermore, the other docked phytocompounds displayed several interactions as detailed in the 2D plot of Figure 5.

Based on the docking outputs described above, these five major phyto‐molecules evaluated *in silico* have significant capabilities to inhibit LOX‐5.

It should be mentioned that molecular docking only offers a theoretical forecast of potential interactions between the target proteins and the discovered chemical. The docking results do not provide direct experimental evidence of the target binding, mechanism, or effect, even though they do indicate the study compounds' possible biological activity. The suggested interactions ought to be regarded as theories that need more verification.

**FIGURE 6:**
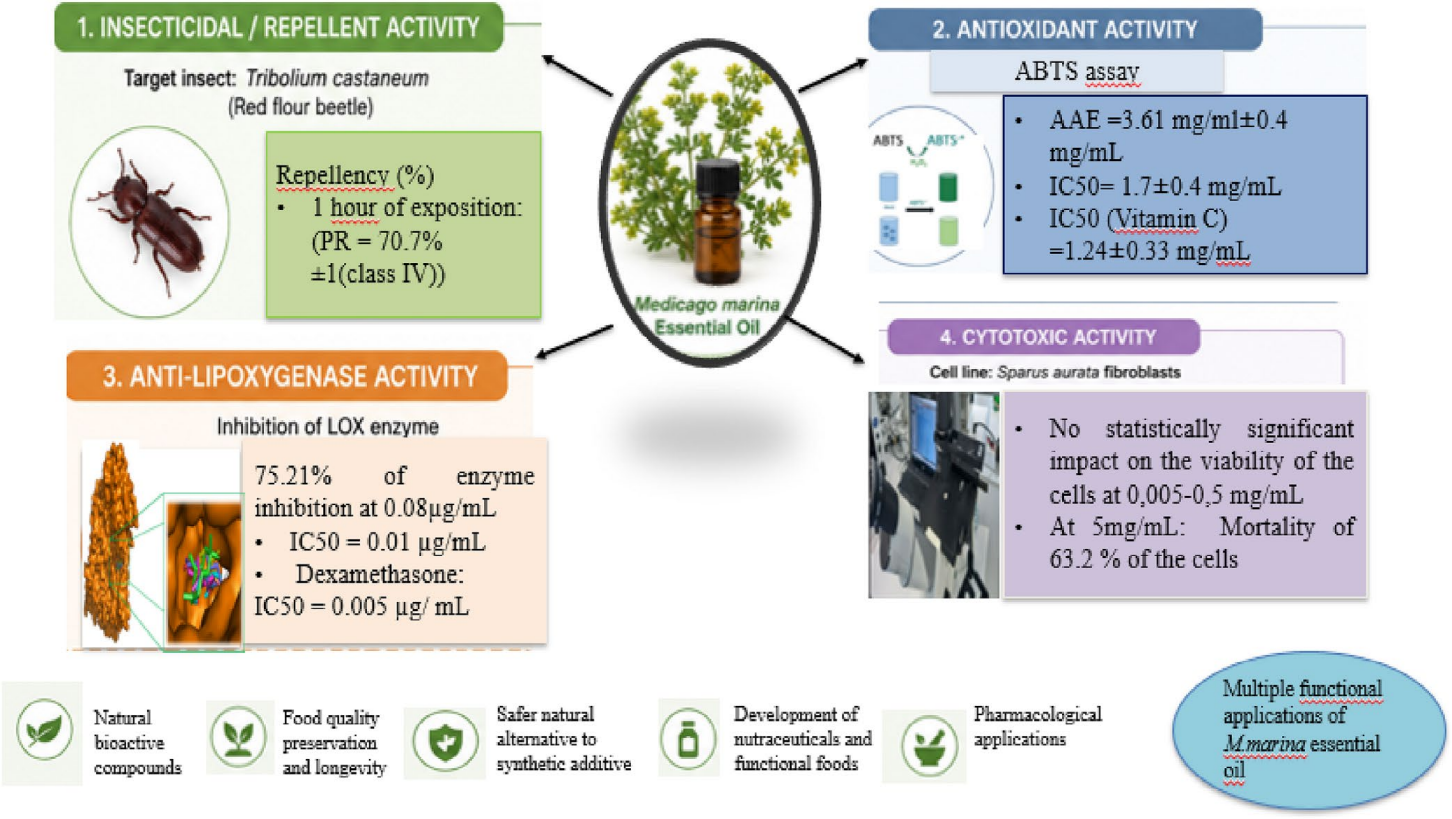
Potential applications of *Medicago marina* essential oil in light of its demonstrated biological activities.

Overall, the results show that the essential oil has a wide range of biological activity, including insecticidal, antioxidant, anti‐lipoxygenase, and cytotoxic activities (Figure 6). These activities should be seen as complementary elements of a chemically complex mixture's biological potential rather than as separate characteristics. While the insecticidal activity emphasizes potential relevance for pest management, the antioxidant and anti‐lipoxygenase activities suggest potential functionality related to oxidative processes and lipid metabolism. On the other hand, the cytotoxicity seen in fish fibroblast cells highlights how crucial it is to assess selectivity and possible impacts on non‐target organisms. Therefore, further mechanistic, in vivo, and application‐oriented research is required to determine the practical value of the essential oil, and its prospective application should be evaluated in connection to both its demonstrated activities and its safety profile.

## 4 Conclusions

In conclusion, the findings of the present study elucidate the diverse properties of *M. marina* EO, including its antioxidant, insecticidal, and anti‐lipoxygenase activities, which were further supported by in silico molecular docking analyses, which predicted favorable binding affinities of the major phytochemicals toward the enzyme's active site compared with the reference compound.

These findings will be instrumental in guiding future investigations into the application of essential oils as additives in fish feed, such as the potential use of this EO in food packaging to deter insects in dry‐food storage. Further research is warranted to determine these bioactive compounds' potential applications in pharmacological and agricultural fields.

## Author Contributions

**Marwa Melliti:** conceptualization, investigation, methodology, visualization, software, formal analysis, data curation, writing – original draft. **Marcello Iriti:** conceptualization, writing – review and editing, supervision, validation, investigation, methodology. **Mabrouk Horchani:** software, methodology. **Ikbel Chaieb:** investigation, writing – original draft, methodology, visualization, software, formal analysis, data curation. **Cristóbal Espinosa‐Ruiz:** investigation, writing – original draft, methodology, visualization, formal analysis, software, data curation. **Maria Angeles Esteban:** conceptualization, supervision, writing – review and editing, validation, investigation, methodology. **Hayet Edziri:** conceptualization, supervision, writing – review and editing, validation, funding acquisition, project administration, resources.

## Funding

This work was supported by the Spanish Government, RYC2023‐045252‐I. European Union Next Generation EU, PRTR‐C17.I1. Comunidad Autónoma de la Región de Murcia‐Fundación Séneca, Spain.

## Disclosure

*Author Approval and Responsibility Statement*: All authors have read and approved the final version of the manuscript. [Marcello Iriti, Maria Angeles Esteban/Hayet Edziri] had full access to all of the data in this study and takes complete responsibility for the integrity of the data and the accuracy of the data analysis.

## Conflicts of Interest

The authors declare no conflicts of interest.

## Data Availability

The data that support the findings of this study are available from the corresponding author upon reasonable request.
